# The measurement of psychological literacy: a first approximation

**DOI:** 10.3389/fpsyg.2015.00105

**Published:** 2015-02-17

**Authors:** Lynne D. Roberts, Brody Heritage, Natalie Gasson

**Affiliations:** School of Psychology and Speech Pathology, Curtin UniversityPerth, WA, Australia

**Keywords:** psychological literacy, undergraduate psychology, measure development, self-report measures, graduate attributes

## Abstract

Psychological literacy, the ability to apply psychological knowledge to personal, family, occupational, community and societal challenges, is promoted as the primary outcome of an undergraduate education in psychology. As the concept of psychological literacy becomes increasingly adopted as the core business of undergraduate psychology training courses world-wide, there is urgent need for the construct to be accurately measured so that student and institutional level progress can be assessed and monitored. Key to the measurement of psychological literacy is determining the underlying factor-structure of psychological literacy. In this paper we provide a first approximation of the measurement of psychological literacy by identifying and evaluating self-report measures for psychological literacy. Multi-item and single-item self-report measures of each of the proposed nine dimensions of psychological literacy were completed by two samples (*N* = 218 and *N* = 381) of undergraduate psychology students at an Australian university. Single and multi-item measures of each dimension were weakly to moderately correlated. Exploratory and confirmatory factor analyses of multi-item measures indicated a higher order three factor solution best represented the construct of psychological literacy. The three factors were reflective processes, generic graduate attributes, and psychology as a helping profession. For the measurement of psychological literacy to progress there is a need to further develop self-report measures and to identify/develop and evaluate objective measures of psychological literacy. Further approximations of the measurement of psychological literacy remain an imperative, given the construct's ties to measuring institutional efficacy in teaching psychology to an undergraduate audience.

## Introduction

The past decade has seen a growing expectation on higher education institutions to prepare their graduates for an increasingly complex, rapidly changing world in which the employees of the future need to be highly-skilled, adaptable, flexible, self-aware, and intuitive problem-solvers with a global outlook. Stakeholders in the tertiary education system (such as students and their families, employers, professional bodies, industry, business, and government) demand greater accountability and clarity about the value of a student's degree and the array of skills they will possess at the completion of their training as they take up a place in the workforce (Cranney et al., 2011a). In the field of psychology, these expectations have led to national and international efforts to define graduate attributes and competencies, student learning outcomes, and career pathways, and to delineate the possibilities/opportunities and boundaries of the discipline of psychology itself. One particular difficulty for the discipline of psychology is that unlike graduates of other health professions (e.g., physiotherapy, occupational therapy), psychology students are not eligible for full professional registration directly upon graduation. This is likely to be contributing to reports from students of psychology that career pathways are not clear (Taylor et al., 2010).

Alongside this has been discussion of the broader skills of university graduates, and how they are prepared to act as “global citizens” with the ability to apply their knowledge to local, national, and international communities in ethical and socially responsible ways for the greater good (Cranney et al., 2011a). Within this context two key constructs in psychology have emerged, the concepts of psychological literacy and the psychologically literate citizen.

The term “psychological literacy” was first used by Boneau (1990) who, in response to a popular activity of the time—defining the core vocabularies of various disciplines—was one of the first researchers to attempt to generate a list of the top 100 concepts/core vocabulary in psychology. He did not specifically define psychological literacy, but referred to his list as a first approximation of psychological literacy, implying that a crucial element of psychological literacy was knowledge of the key terms and concepts. Interest in the core concepts and accurate premises and understanding of psychology had actually been present in the literature since the mid-1920s, with the emergence of psychological misconceptions research (which could be viewed as a precursor to the more complex and detailed analysis of the construct of psychological literacy that is now emerging). There were scattered pieces of research on myths and misconceptions in psychology up until the influential work of Vaughan (1977) who developed the Test of Common Beliefs (TCB). This led to an increase in research on misconceptions in psychology which has continued to the present (see Hughes et al., 2013b for a recent review). Following from Boneau's reference to psychological literacy, the concept of psychological literacy was next discussed (but not defined) by O'Hara (2007) in relation to the demands of the changing modern world, globalization, and the potential role psychologists could perform for the common benefit of people faced with psychological distress and crisis. O'Hara theorized that just as technological literacy was advancing, we would also need advanced psychological literacy to respond to the increasing complexities and challenges of the world around us.

The most substantial, well-accepted, and heavily referenced definition of psychological literacy to date was proposed by McGovern et al. (2010) who linked psychological literacy to the universal attributes that graduates from psychology degrees should display. The authors stated that psychological literacy means:

- having a well-defined vocabulary and basic knowledge of the critical subject matter of psychology;
- valuing the intellectual challenges required to use scientific thinking and the disciplined analysis of information to evaluate alternative courses of action;
- taking a creative and amiable skeptical approach to problem solving;
- applying psychological principles to personal, social, and organizational issues in work, relationships, and the broader community;
- acting ethically;
- being competent in using and evaluating information and technology;
- communicating effectively in different modes and with many different audiences;
- recognizing, understanding, and fostering respect for diversity;
- being insightful and reflective about one's own and others' behavior and mental processes. (p. 11)

The McGovern et al. definition has been described as a comprehensive and useful conceptualization of psychological literacy, and although it is quite detailed, this is seen by some as necessary to convey an essentially complex and dense construct (Beins et al., 2011; Halpern and Butler, 2011; Cranney et al., 2011b, 2012). This definition also maps closely to the Australian Psychology Accreditation Council (APAC) graduate attributes as outlined in the Accreditation Standards (Australian Psychology Accreditation Council, 2010). These are: core knowledge and understanding; research methods in psychology; critical thinking skills; values, research and professional ethics; communication skills; and learning and the application of psychology (Australian Psychology Accreditation Council, 2010, pp. 41–42).

An alternative, broader definition was provided by Cranney et al. (2012) who proposed that psychological literacy is “… the general capacity to adaptively and intentionally apply psychology to meet personal, professional, and societal needs” (p. iii). This definition reflects the more aspirational approach to psychological literacy held by Cranney et al. who has been at the forefront of recent research in psychological literacy in Australia. Cranney, along with other researchers, promotes psychological literacy as a necessary goal and primary outcome of an undergraduate education in psychology (McGovern et al., 2010; Beins et al., 2011; Halpern and Butler, 2011; Cranney et al., 2011a,b). Cranney and Morris (2011) have extended the conceptualization of psychological literacy with the introduction of the idea of adaptive cognition—“… global ways of thinking (and consequently behaving) that are beneficial to one's (and others') survival and wellbeing” (2011, p. 251). In this conceptualization of psychological literacy, while all students who undertake some study of psychology may develop a level of psychological literacy required to act as psychologically literate citizens, graduates of psychology are seen as uniquely placed to apply their store of psychological knowledge to solve not only the personal, family, and local community challenges encountered in the modern world, but to have the potential to influence and problem-solve at a national and international level for the benefit of others (i.e., acting as psychologically literate citizens).

A review of the two dominant conceptualizations of psychological literacy presented here reveals a complex construct that embodies everything from the core knowledge and concepts learnt at an introductory psychology level, through to scientific literacy (including understanding of the scientific method and principles of research), skills of critical thinking, oral and written communication skills, knowledge of ethics and diversity, self-awareness and self-reflection, and the ability to apply all of this in a range of ways to a wide variety of situations. As stated by Bernstein, the conceptualization of psychological literacy as presented by McGovern et al. (2010) is “broad, sweeping, and contemporary” (2011, p. 281). While accepting of psychological literacy, its value as a construct, and its inherent complexity, Beins et al. (2011) highlight the need for a more precise operationalisation of psychological literacy if research is to move forward. There is urgent need to define the boundaries, link the construct with how it will be measured, and begin to investigate if what we measure as “psychological literacy” has real-world application and utility. One of the key questions is—will it be a multi-faceted construct with multiple measures, or will there be one “grand scale” of psychological literacy? (Beins et al., 2011). As the concept of psychological literacy becomes increasingly adopted as the core business of undergraduate psychology training courses world-wide, there is urgent need for the construct to be accurately measured so that student and institutional level progress can be assessed and monitored, with some authors calling for a standardized assessment to be developed as soon as possible (Halpern and Butler, 2011).

To date, published psychological literacy research has used single-item self-report measures of overall psychological literacy (e.g., “How developed is your own psychological literacy?”; Morris et al., 2013), or single-item measures for each of the psychological literacy dimensions (e.g., “At this point in your education, how would you rate your knowledge of basic concepts/principles in Psychology?”; Chester et al., 2013). Single item measures of multi-dimensional abstract constructs, such as psychological literacy, are not recommended, as their complexity cannot be captured in a single item and they are prone to high measurement error (Fuchs and Diamantopoulos, 2009). Single item measures of psychological literacy dimensions (assuming each dimension represents a concrete, single factor) may be more defensible, but before being accepted as adequate for research purposes, reliability and validity need to be determined (see Fuchs and Diamantopoulos, 2009 for an overview of ways of assessing reliability and validity). No reliability and validity information has currently been provided for the single item measures of psychological literacy.

## The current study

Our research aims to build on that of Beins et al. (2011) who recommend that researchers begin the process of identifying and evaluating measures of psychological literacy dimensions. In addition to the single item measures of psychological literacy dimensions (Chester et al., 2013), a search of the literature identified psychometrically sound measures that may capture the essence of each of the nine facets of psychological literacy outlined by McGovern et al. (2010). The first step in determining whether the measurement of psychological literacy requires a single or multiple measures is to determine the level of association between measures of the differing facets of psychological literacy. Non-significant or low correlations between measures would indicate that multiple measures are required to measure psychological literacy. High correlations would provide support for the future development of a unitary measure of psychological literacy. Correlations mixed in magnitude may indicate the possibility of a higher order factor structure. The second step is to examine the factor structure underlying the measures. As this is exploratory research, exploratory factor analysis is suitable for this purpose. The third step is to determine whether the factor structure can be replicated in a new sample. Confirmatory factor analysis is suitable for this purpose.

The identification of measures for each psychological literacy dimension also provides the opportunity to begin the evaluation of the single item measures of psychological literacy dimensions (Chester et al., 2013). Moderate to high correlations between the single item measure and multiple-item measure of each psychological literacy dimension would be expected if the measures are capturing the same construct.

## Method

### Participants

The participants for this research were convenience samples of 218 (phase 1) and 381 (phase 2) psychology students at an Australian university. Phase one participants (age *M* = 22.69 years, *SD* = 7.52 years) included 48 male and 170 female students. Phase two participants (age *M* = 20.51 years, *SD* = 5.16 years) included 95 male and 286 female students. Demographic characteristics are presented in Table 1 for both phase one and phase two participants. Participants were most commonly from the first and second year undergraduate cohorts of the university's undergraduate psychology programs. The majority of students were enrolled in full-time study on a domestic basis.

**Table 1:** **Demographic characteristics of phase one and phase two participants**.

	**Phase One (*N* = 218)**	**Phase Two (*N* = 381)**
**GENDER**		
Male	48	95
Female	170	286
**YEAR OF STUDY**		
First year undergraduate	35	187
Second year undergraduate	144	166
Third year undergraduate	16	25
Fourth year undergraduate	17	2
Postgraduate	6	1
**ENROLMENT^a^**		
Full-time	178	329
Part-time	38	50
**REGISTRATION^b^**		
Domestic students	208	364
International students	9	17

a *Two students did not provide information on their enrolment status*.

b *One student did not provide information on their registration status*.

### Measures

An online questionnaire was developed comprising single-item and multi-item measures of the nine facets of psychological literacy, followed by demographic items (age, gender, years of study, number of psychology units completed, full time or part-time status, international or domestic student). Each measure was presented on a separate page (or across two or three pages for longer measures) with a maximum of 10 items per page. Items for each measure were displayed in a matrix to allow ease of responding.

#### Single item measures of psychological literacy dimensions (Chester et al., 2013)

Nine single items asked students to self-rate their competencies against each of the nine psychological literacy dimensions using a four-point Likert scale response option: 1 (non-existent), 2 (poor), 3 (reasonable), 4 (excellent). The example item provided by Chester et al. (2013) is “At this point in your education, how would you rate your knowledge of basic concepts/principles in Psychology?.” Previous research with first year students indicated that scores on each of the items (excluding self-awareness) increased following an 8 week peer mentoring intervention (Chester et al., 2013). No further reliability or validity information is available for these items.

#### Psychology Misconceptions Test (Hughes et al., 2013a)

The Psychology Misconceptions Test was used to measure the first facet of psychological literacy: having a well-defined vocabulary and basic knowledge of the critical subject matter of psychology. The test consists of 29 statements that are common misconceptions about psychology. An example item is “People predominantly use either the left side or the right side of their brain.” Participants respond to each item with “True” “False” or “Uncertain.” This test has known groups validity, with higher test scores with increasing years of study, and psychology students performing better than non-psychology students (Hughes et al., 2013a).

#### Need for Cognition Scale (short form; Cacioppo et al., 1984)

The Need for Cognition Scale was used to measure the second facet of psychological literacy: valuing the intellectual challenges required to use scientific thinking and the disciplined analysis of information to evaluate alternative courses of action. The Need for Cognition Scale (short form) has 18 items with a nine-point Likert scale anchored by “strongly disagree” and “strongly agree.” An example item is “I find satisfaction in deliberating hard and for long hours.” All items load on one factor. Scores on the short form are highly correlated with scores of the full 34 item measure (*r* = 0.95). The scale is unidimensional and has good internal reliability (α = 0.90; Cacioppo et al., 1984).

#### Critical Thinking Disposition Scale (Sosu, 2013)

The Critical Thinking Disposition Scale was used to measure the third facet of psychological literacy: taking a creative and amiable skeptical approach to problem solving. This measure consists of 11 items that are responded to on a five-point Likert scale (1 = strongly disagree, 5 = strongly agree). Example items are “It is important to justify the choices I make” (critical openness) and “I usually check the credibility of the source of information before making judgements” (reflective skepticism). A combination of exploratory and confirmatory factor analysis indicate that there are two factors underlying the measure, reflective skepticism and critical openness (Sosu, 2013). Designed for use as a single measure, the combined scale has good internal reliability (α = 0.79–0.81; Sosu, 2013).

#### Psychology as a Helping Profession Scale (Gervasio et al., 2010)

The Psychology as a Helping Profession Scale was developed to measure “beliefs about helping-related content and skills found in undergraduate psychology courses” (Gervasio et al., 2010). This scale was used to measure the fourth facet of psychological literacy: applying psychological principles to personal, social, and organizational issues in work, relationships, and the broader community. This measure consists of 11 items with Likert scale response options of 1 (strongly disagree) to 7 (strongly agree). An example item is “People can learn to enhance their health (e.g., stop smoking) through courses in psychology.” Two factors, reflecting personal growth and applied helping, underlie the measure. Designed for use as a single measure, the combined scale has good internal reliability (α = 0.82). The measure has known groups validity: psychology students score higher than non-psychology students (Gervasio et al., 2010).

#### The Integrity Scale (Schlenker, 2008)

The Integrity Scale was used to measure the fifth facet of psychological literacy: acting ethically. This measure consists of 18 items with Likert scale response options ranging from 1 (strongly disagree) to 5 (strongly agree). An example item is “It is important to me to feel that I have not compromised my principles.” The scale is unidimensional, has good internal consistency (α = 0.84–0.90) and test-retest reliability at 2–5 week (*r* = 0.82) and 5–12 week (*r* = 0.72) intervals (Schlenker, 2008).

#### Information Literacy Self-Efficacy Scale (Kurbanoglu et al., 2006)

The 17 item version of the Information Literacy Self-Efficacy Scale was used to measure the sixth facet of psychological literacy: being competent in using and evaluating information and technology. This scale consists of 17 items with a seven-point Likert scale response option ranging from 7 almost always true to 1 almost never true. An example item is “Locate resources in the library using the library catalog.” Exploratory factor analysis indicates that three factors; basic, intermediate, and advanced information literacy skills; underlie the measure. Designed for use as a single measure, the combined scale has good internal reliability (α = 0.82; Kurbanoglu et al., 2006).

#### Self-perceived Communication Competence Scale (Mccroskey and Mccroskey, 1988)

The Self-perceived Communication Competence Scale was used to measure the seventh facet of psychological literacy: communicating effectively in different modes and with many different audiences. The measure consists of 12 items that address communicating in public, in meetings, in groups and in dyads, and with strangers, acquaintances and friends. An example item is “Present a talk to a group of strangers.” For each item, respondents rate their competence on a scale from 0 to 100. A total score can be computed, and has high internal reliability (α = 0.92; McCroskey and McCroskey, 1988).

#### Interactional Diversity Scale (Hu and Kuh, 2003)

The Interactional Diversity Scale was used to measure the eighth facet of psychological literacy: recognizing, understanding, and fostering respect for diversity. The scale consists of seven items with Likert scale response options of 1 (never) to 4 (very often). An example item is “Had serious discussions with students whose religious beliefs were very different from yours.” The measure has high internal reliability (α = 0.89; Hu and Kuh, 2003).

#### Self-Reflection and Insight Scale (Grant et al., 2002)

An extended version of the Self-Reflection and Insight Scale was used to measure the ninth facet of psychological literacy: being insightful and reflective about one's own and others' behavior and mental processes. The original 20 items measure one's own behavior and mental processes only, using a six-point Likert scale response option ranging from 1 (strongly disagree) to 6 (strongly agree). Items load onto two factors: insight and self-reflection. Example items are “I frequently examine my feelings” (self-reflection scale) and “I usually know why I feel the way I do” (insight scale). The scales have good internal reliability (0.71–0.91 across two studies; Grant et al., 2002). Because this scale measures insight and reflection to own, and not others' behavior, six new items have been developed to measure this. The items were modeled on individual insight and reflection items and are:

I don't really think about why others behave in the way that they do (R).
I am not really interested in analysing other people's behavior (R).
It is important for me to evaluate the things that other people do.
It is important to me to try to understand what other people's feelings mean.
I have a definite need to understand the way that other people's minds work.
Other people's behavior often puzzles me (R).

### Procedure

Following Curtin University Human Research Ethics Committee approval, recruitment for the study was conducted in two time periods: the first semester of 2013 and 2014. The study was advertised at the beginning of psychology lectures and through postings on a learning management system accessible by all undergraduate psychology students. Psychology students were also recruited from the School's research participation pool, with participating students awarded research points for participation. Other participating students were entered into a prize draw for a $100 Amazon.com voucher.

The online questionnaire was developed and hosted on the Qualtrics website. The questionnaire was “sandwiched” between a participant information sheet and a debriefing page hosted on the School website in line with best practice recommendations (Allen and Roberts, 2010). Interested students were provided with a link to the participant information sheet and upon consenting to participate were redirected to the questionnaire. The majority of students completed the survey within 15–40 min. Survey data were downloaded from Qualtrics into SPSS (v. 20) for analysis. The data was screened for multiple responding and missing values. Cases that missed at least an entire subscale were listwise deleted, and only cases who were enrolled in a psychology degree were retained for analysis. This resulted in samples of *N* = 218 for Phase 1 and *N* = 381 for Phase 2.

## Results

### Phase one data

Prior to conducting analyses to test the relatedness of the facets of psychological literacy, the factor structure of each scale measure was examined using confirmatory factor analysis (CFA; see Table 2) to examine whether a one-factor model was sufficient to describe the factor structure of each measure, and the internal reliability determined using Cronbach's alpha (see Table 3). CFA and internal reliability tests were not conducted for the Psychology Misconceptions Test as this is a knowledge test rather than a scale measure. The model adequacy coefficients indicated deviations from good fit for each measure. However, all internal consistencies were adequate for the phase one data, with the Insight subscale of the Self-Reflection and Insight Scale presenting the lowest Cronbach's alpha coefficient of the chosen measures, α = 0.68. Scale sores were computed using factor loadings from the CFAs.

**Table 2:** **Summary of model adequacy coefficients for confirmatory factor analyses conducted to derive factor loadings on measures of psychological literacy**.

	**χ^2^ (*df*)**	***CFI***	***TLI***	***RMSEA***	***SRMR***
**PHASE ONE (*N* = 218)**					
CTDS	109.82 (44)^***^	0.843	0.804	0.083	0.066
Integrity	467.14 (136)^***^	0.649	0.605	0.106	0.091
I. Diversity	132.98 (14)^***^	0.875	0.813	0.197	0.070
NFC	403.04 (135)^***^	0.751	0.717	0.095	0.092
PHP	118.71 (43)^***^	0.883	0.851	0.090	0.072
SPCC	326.50 (54)^***^	0.781	0.733	0.152	0.099
SRIS	847.34 (298)^***^	0.784	0.765	0.092	0.122
ILSES	706.52 (119)^***^	0.698	0.655	0.150	0.095
**PHASE TWO (*N* = 381)**					
CTDS	200.25 (44)^***^	0.786	0.732	0.097	0.070
Integrity	571.12 (136)^***^	0.560	0.505	0.092	0.090
I. Diversity	231.38 (14)^***^	0.849	0.774	0.202	0.077
NFC	543.86 (135)^***^	0.795	0.767	0.089	0.071
PHP	154.61 (43)^***^	0.897	0.869	0.083	0.055
SPCC	174.66 (54)^***^	0.818	0.777	0.077	0.096
SRIS	1186.22 (298)^***^	0.779	0.759	0.088	0.106
ILSES	1032.775 (119)^***^	0.692	0.648	0.142	0.095

*CTDS, Critical Thinking Disposition Scale; Integrity, Integrity Scale; I. Diversity, Interaction Diversity Scale; NFC, Need for Cognition Scale; PHP, Psychology as a Helping Profession; SPCC, Self-Perceived Communication Competence Scale; SRIS, Self-Reflection and Insight Scale; ILSES, Information Literacy Self Efficacy Scale; χ^2^ (df), Robust chi-square test (degrees of freedom); CFI, Comparative Fit Index; TLI, Tucker-Lewis Fit Index; RMSEA, Root Mean Square Error of Approximation; SRMR, Standardised Root Mean Square Residual*.

*Cut-off criteria for acceptable model fit per recommendations in Byrne (2012): CFI/TLI >0.95; RMSEA <0.08; SRMR <0.05*.

*** *p < 0.001*.

**Table 3:** **Pearson correlation coefficients and internal consistencies of scales for each facet of psychological literacy in phase one data (*N* = 218)**.

	**CTDS**	**Integrity**	**Diversity**	**NFC**	**PHP (F1)**	**PHP (F2)**	**SPCC**	**SRIS (SR)**	**SRIS (IN)**	**SRIS (OT)**	**ILSES**	**PMT**
CTDS	0.802											
Integrity	0.366^**^	0.825										
Diversity	0.202^**^	0.193^**^	0.906									
NFC	0.459^**^	0.227^**^	0.051	0.878								
PHP (F1)	0.236^**^	0.221^**^	0.061	0.121	0.807							
PHP (F2)	0.125	0.168^*^	−0.007	−0.023	0.685^**^	0.786						
SPCC	0.286^**^	0.175^**^	0.160^*^	0.315^**^	0.068	0.011	0.927					
SRIS (SR)	0.551^**^	0.273^**^	0.179^**^	0.405^**^	0.298^**^	0.124	0.115	0.909				
SRIS (IN)	0.231^**^	0.294^**^	−0.008	0.384^**^	0.079	−0.036	0.314^**^	0.292^**^	0.683			
SRIS (OT)	0.412^**^	0.254^**^	0.063	0.325^**^	0.307^**^	0.170^*^	0.100	0.736^**^	0.283^**^	0.737		
ILSES	0.274^**^	0.273^**^	0.109	0.357^**^	0.224^**^	0.061	0.463^**^	0.182^**^	0.406^**^	0.168^*^	0.933	
PMT	0.236^**^	0.089	−0.075	0.299^**^	0.017	−0.046	0.210^**^	0.187^**^	0.166^*^	0.151^*^	0.286^**^	0.794
*M*	4.03	3.62	2.68	5.99	5.03	5.07	73.54	4.83	3.99	4.59	5.40	37.80
*SD*	0.45	0.48	0.74	1.07	0.92	0.94	15.94	0.74	0.68	0.68	0.90	11.08

*Internal consistencies (α) are represented along the diagonal, and are calculated via raw data. Pearson correlations are based on factor loading scores for each scale/subscale. CTDS, Critical Thinking Disposition Scale; Integrity, Integrity Scale; Diversity, Interaction Diversity Scale; NFC, Need for Cognition Scale; PHP (F1), Psychology as a Helping Profession (Factor One); PHP (F2), Psychology as a Helping Profession (Factor Two); SPCC, Self-Perceived Communication Competence Scale; SRIS (SR), Self-Reflection and Insight Scale (Self-Reflection Subscale); SRIS (IN), Self-Reflection and Insight Scale (Insight Subscale); SRIS (OT), Self-Reflection and Insight Scale (Others Reflection Subscale); ILSES, Information Literacy Self Efficacy Scale; PMT, Psychology Misconceptions Test (False Responses)*.

** *p < 0.01*.

* *p < 0.05*.

The first step in determining whether the measurement of psychological literacy requires a single or multiple measures was to determine the level of association between measures of the differing facets of psychological literacy. Examination of correlations between factor loadings indicated two potential multicollinearity concerns for the planned Exploratory Factor Analysis (EFA); the Critical Openness subscale and Reflective Skepticism subscale of the Critical Thinking Disposition Scale were highly correlated (*r* = 0.86, *p* < 0.001), as were the Advanced and Intermediate subscales of the Information Literacy Self Efficacy scale (*r* = 0.94, *p* < 0.001). In order to maintain construct validity, factor scores for all items on the Critical Thinking Disposition Scale and the Information Literacy Self Efficacy Scale were recalculated using unidimensional factor loadings. Multicollinearity issues were not present among the factor scores for each scale following this procedure (see Table 3).

Inspection of the correlation matrix indicates that correlations between measures range from non-significant to moderate. The highest correlations were found between subscales of the same measure (e.g., the two subscales of Psychology as a Helping Profession). As noted in the introduction, correlations mixed in magnitude may indicate the possibility of a higher order factor structure, and this was then examined through EFA of factor scores.

Horn's (1965) parallel analysis method of determining interpretable factors derived from EFA was conducted, indicating three factors should be extracted. EFA with Promax (oblique) rotation was employed to account for the predicted correlations between latent factors of psychological literacy. Overall, the extracted model represented a good fit to the data, χ^2^_(*S*−*B*)_ (*N* = 218, *df* = 33) = 0.131, *p* = 1.000 (see Table 4 for indicator loadings).

**Table 4:** **Rotated exploratory factor loadings of measures of psychological literacy, using phase one data (*N* = 218)**.

	**RP**	**GGA**	**PHP**
Self-Reflection and Insight Scale (Self-Reflection Subscale)	1.041		
Self-Reflection and Insight Scale (Others Reflection Subscale)	0.767		
Critical Thinking Disposition Scale	0.428		
Information Literacy Self Efficacy Scale		0.792	
Self-Perceived Communication Competence Scale		0.698	
Self-Reflection and Insight Scale (Insight Subscale)		0.522	
Need for Cognition Scale		0.494	
Psychology Misconceptions Test (False Responses)		0.369	
Integrity Scale		0.330	
Psychology as a Helping Profession (Factor Two)			0.834
Psychology as a Helping Profession (Factor One)			0.826
Interactional Diversity Scale			

*MLM robust extraction method with Promax rotation used to extract factor loadings; Loadings < 0.300 are suppressed in the table for clarity; Model χ^2^_(S–B)_ (N = 218, df = 33) = 0.131, p = 1.000; RP, Reflective Processes; GGA, Generic Graduate Attributes; PHP, Psychology as a Helping Profession*.

The first extracted factor consisted of the Self-Reflection and Others' Reflection subscales of the Self-Reflection and Insight Scale, and the Critical Thinking Disposition Scale. These measures collectively suggested a latent factor representative of *Reflective Processes*. The second extracted factor spanned the largest quantity of facets of psychological literacy, and was composed of the Information Literacy Self-Efficacy Scale, the Self-Perceived Communication Competence Scale, the Insight subscale of the Self-Reflection and Insight Scale, the Need for Cognition Scale, the Psychological Misconception Test, and the Integrity Scale. These scales suggested a latent factor representative of broader *Generic Graduate Attributes* for students engaged in tertiary education, with the exception of the Psychological Misconception Test which is of a more discipline-specific nature. The third extracted factor consisted of strong loadings from both subscales of the *Psychology as a Helping Profession Scale*. The Interactional Diversity Scale did not load meaningfully on any of the extracted factors. Correlations between latent factors varied in strength, with the first two factors (Reflective Processes and Graduate Attributes) being moderately correlated (*r* = 0.53), while Reflective Processes and Psychology as a Helping Profession (*r* = 0.27) and Graduate Attributes and Psychology as a Helping Profession (*r* = 0.13) were more weakly correlated.

### Phase two data

In order to examine whether the factor structure of the indicators of psychological literacy based on the Phase one data was replicable when applied to a different sample, CFA was conducted on the data from 381 participants from Phase two. Mplus' MLM (robust maximum-likelihood) estimator was employed as the method of model estimation to account for potential influences of non-normality. MLM has demonstrated accuracy in model estimation when the indicator quantity per factor can be considered as ordered categorical data (four to six response options) (Green et al., 1997), and tests of competing estimation procedures (ML, WLSMV in the case of response scales with less than seven options) did not produce meaningful differences in model interpretation. The specified model provided poor fit to Phase 2 sample data (see Table 5 for item loadings, standard errors and fit statistics).

**Table 5:** **Standardised factor loadings and standard errors for confirmatory model of psychological literacy from phase two data (*N* = 381)**.

	**RP**	**GGA**	**PHP**
Self-Reflection and Insight Scale (Self-Reflection)	0.704 (0.065)		
Self-Reflection and Insight Scale (Others Reflection)	0.634 (0.048)		
Critical Thinking Disposition Scale	0.777 (0.038)		
Information Literacy Self Efficacy Scale		0.548 (0.045)	
Self-Perceived Communication Competence Scale		0.329 (0.059)	
Self-Reflection and Insight Scale (Insight Subscale)		0.315 (0.055)	
Need for Cognition Scale		0.742 (0.042)	
Psychology Misconceptions Test (False Responses)		0.364 (0.055)	
Integrity Scale		0.356 (0.054)	
Psychology as a Helping Profession (Factor Two)			0.748 (0.057)
Psychology as a Helping Profession (Factor One)			0.889 (0.064)

*Standard errors provided in brackets; MLM robust estimation procedures used; Model χ^2^_(S-B)_ (N = 381, df = 41) = 153.14, p < 0.001; CFI = 0.861; TLI = 0.813; RMSEA = 0.085; SRMR = 0.062; RP, Reflective Processes; GGA, Generic Graduate Attributes; PHP, Psychology as a Helping Profession*.

In light of the poor model fit, EFA was conducted on the Phase 2 data, as it is considered preferable to perusing modification indices and engaging in exploratory elements of CFA (Browne, 2001). Correlations between factors and internal consistencies are presented in Table 6. Parallel analysis again indicated a three-factor solution was most appropriate for extraction for the EFA. The extracted model had good model fit, χ^2^_(*S*−*B*)_ (*N* = 381, *df* = 33) = 0.128, *p* = 1.000 (see Table 7 for factor loadings).

**Table 6:** **Pearson correlation coefficients and internal consistencies of scales representative of the facets of psychological literacy in phase two sample (*N* = 381)**.

	**CTDS**	**Integrity**	**Diversity**	**NFC**	**PHP (F1)**	**PHP (F2)**	**SPCC**	**SRIS (SR)**	**SRIS (IN)**	**SRIS (OT)**	**ILSES**	**PMT**
CTDS	0.802											
Integrity	0.351^**^	0.737										
Diversity	0.184^**^	0.123^*^	0.896									
NFC	0.512^**^	0.293^**^	0.198^**^	0.885								
PHP (F1)	0.318^**^	0.135^**^	0.056	0.125^*^	0.817							
PHP (F2)	0.328^**^	0.205^**^	0.117^*^	0.123^*^	0.665^**^	0.802						
SPCC	0.206^**^	0.005	0.217^**^	0.229^**^	0.072	−0.002	0.920					
SRIS (SR)	0.495^**^	0.164^**^	0.107^*^	0.345^**^	0.298^**^	0.193^**^	0.020	0.917				
SRIS (IN)	0.201^**^	0.012	0.001	0.184^**^	0.064	0.052	0.212^**^	0.150^**^	0.627			
SRIS (OT)	0.442^**^	0.096	0.051	0.241^**^	0.240^**^	0.178^**^	0.097	0.612^**^	0.119^*^	0.712		
ILSES	0.383^**^	0.189^**^	0.129^*^	0.391^**^	0.133^**^	0.137^**^	0.303^**^	0.194^**^	0.176^**^	0.141^**^	0.922	
PMT	0.169^**^	0.077	−0.045	0.278^**^	−0.091	−0.016	0.104^*^	0.120^*^	0.291^**^	0.100	0.203^**^	0.776
*M*	3.97	3.52	2.75	5.77	5.06	5.09	72.65	4.76	3.86	4.51	5.31	17.10
*SD*	0.47	0.41	0.73	1.12	0.91	0.93	15.93	0.78	0.63	0.67	0.86	5.35

*Internal Consistencies (a) are represented along the diagonal, and are calculated via raw data. Pearson correlations are based on factor loading scores for each scale/subscale. CTDS, Critical Thinking Disposition Scale; Integrity, Integrity Scale; Diversity, Interaction Diversity Scale; NFC, Need for Cognition Scale; PHP (F1), Psychology as a Helping Profession (Factor One); PHP (F2), Psychology as a Helping Profession (Factor Two); SPCC, Self-Perceived Communication Competence Scale; SRIS (SR), Self-Reflection and Insight Scale (Self-Reflection Subscale); SRIS (IN), Self-Reflection and Insight Scale (Insight Subscale); SRIS (OT), Self-Reflection and Insight Scale (Others Reflection Subscale); ILSES, Information Literacy Self Efficacy Scale; PMT, Psychology Misconceptions Test (False Responses)*.

** *p < 0.01*.

* *p < 0.05*.

**Table 7:** **Rotated exploratory factor loadings of measures of psychological literacy, using phase two data (*N* = 381)**.

	**GGA**	**RP**	**PHP**
Need for Cognition Scale	0.687		
Information Literacy Self Efficacy Scale	0.597		
Critical Thinking Disposition Scale	0.553		
Self-Perceived Communication Competence Scale	0.441		
Psychology Misconceptions Test (False Responses)	0.375		
Integrity Scale	0.343		
Self-Reflection and Insight Scale (Insight Subscale)	0.301		
Self-Reflection and Insight Scale (Self-Reflection Subscale)		0.838	
Self-Reflection and Insight Scale (Others Reflection Subscale)		0.689	
Psychology as a Helping Profession (Factor Two)			0.911
Psychology as a Helping Profession (Factor One)			0.724
Interactional Diversity Scale			

*MLM robust extraction method with Promax rotation used to extract factor loadings; Loadings <0.300 are suppressed in the table for clarity; Model χ^2^_(S-B)_ (N = 381, df = 33) = 0.128, p = 1.000; RP, Reflective Processes; GGA, Generic Graduate Attributes; PHP, Psychology as a Helping Profession*.

**Figure 1:**
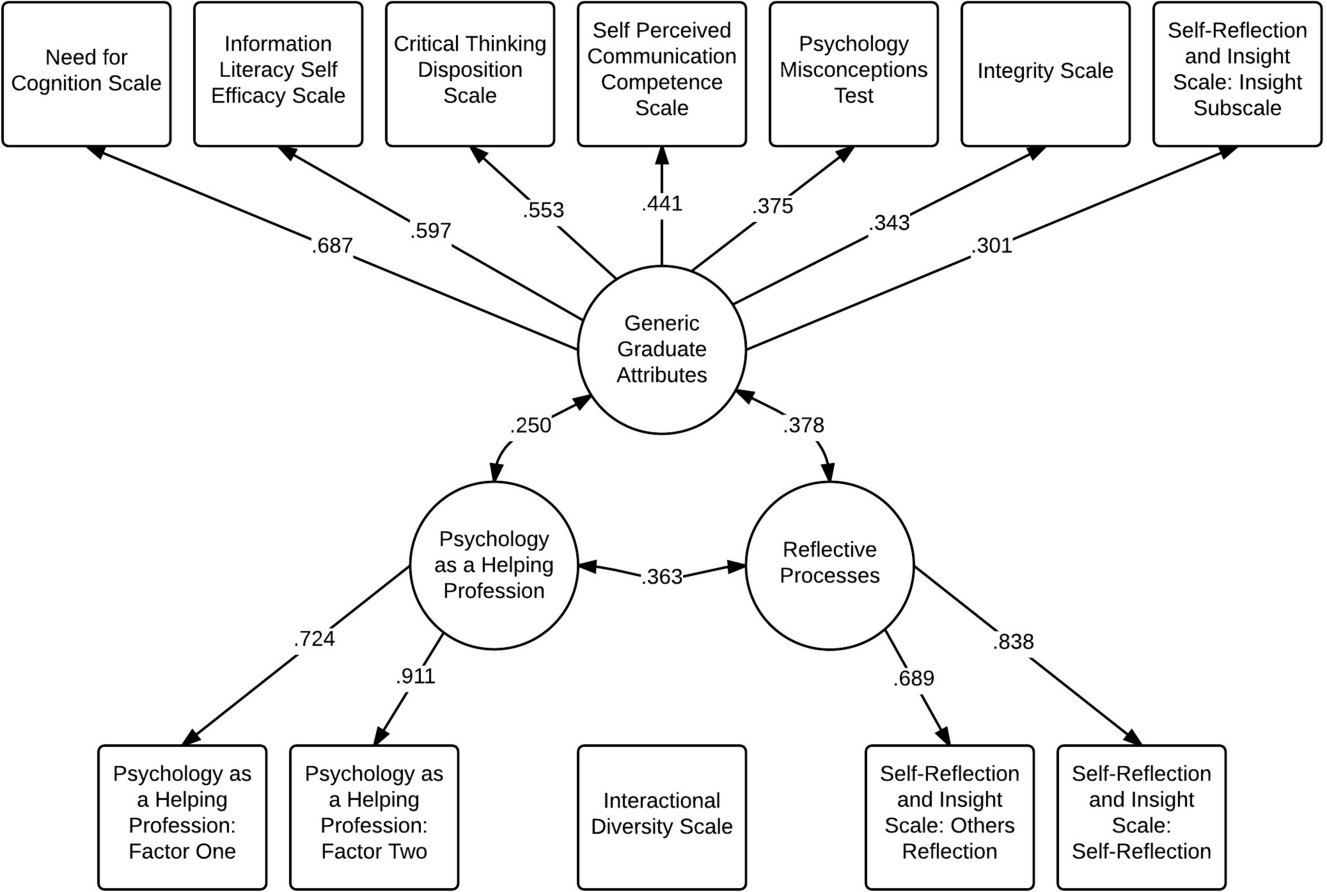
**Factor structure of psychological literacy**.

A thematically-similar pattern of indicators on extracted latent factors emerged in the follow-up EFA. The first extracted factor appeared to be representative of broader graduate attributes again, as it consisted of the Information Literacy Self Efficacy Scale, the Need for Cognition Scale, the Critical Thinking Disposition Scale, the Self Perceived Communication Competency Scale, the Psychology Misconceptions Test, the Integrity Scale, and the Insight subscale of the Self-Reflection and Insight Scale. The second extracted factor appeared to be representative of reflective processes again, as it was composed of the Self-Reflection and Others Reflection subscales of the Self-Reflection and Insight Scale. The third extracted factor mirrored the results of the Phase 1 data EFA, consisting of the *Psychology as a Helping Profession* subscales. The Interactional Diversity Scale did not load on any of the extracted factors, mirroring the results of the first phase's EFA outcomes. Small to moderate correlations between extracted factors were presented, with Graduate Attributes and Reflective Practices (*r* = 0.38), Graduate Attributes and Psychology as a Helping Profession (*r* = 0.25), and Reflective Processes and Psychology as a Helping Profession (*r* = 0.36). The final model is depicted in Figure 1.

To examine whether single-item and multiple-item measures of each facet of psychological literacy were capturing the same constructs, correlations between each pair of measures were analyzed (see Table 8). Phase one and two data demonstrated similar magnitudes of relationships between single- and multiple-item measures. The information literacy and communication competency correlations demonstrated moderate relationships between their single- and multiple-item measures. However, the remaining scales demonstrated small or non-significant relationships between the single- and multiple-item measures. The absence of strong correlations (*r* > 0.70) between single and multi-item measures of each psychological literacy facet indicates that they are not measuring the same constructs and cannot be used interchangeably.

**Table 8:** **Bivariate correlation coefficients (*r*) between multiple-item and single-item predictors of the facets of psychological literacy**.

	**Phase one**	**Phase two**
Knowledge of basic psychology concepts/principles	0.153^*^	0.100
Valuing intellectual challenge / evaluating actions	0.129	0.155^**^
Creative/skeptical problem solving	0.172^*^	0.146^**^
Applying psychology principles to issues in multiple contexts (F1)	0.051	0.019
Applying psychology principles to issues in multiple contexts (F2)	−0.035	0.050
Acting ethically	0.157^*^	0.120
Ability to use and access information	0.520^**^	0.462^**^
Communicate effectively in different modes/with different audiences	0.401^**^	0.490^**^
Recognise and respect diversity	0.242^**^	0.188^**^
Having insight/reflection on own/others' behaviors and processes (SR)	0.347^**^	0.277^**^
Having insight/reflection on own/others' behaviors and processes (IN)	0.224^**^	0.204^**^
Having insight/reflection on own/others' behaviors and processes (OT)	0.296^**^	0.232^**^

*F1, First factor of the Psychology as a Helping Profession Scale correlated with the respective single-item measure; F2, Second factor of the Psychology as a Helping Profession Scale correlated with the respective single-item measure; SR, Self-reflection subscale of the Self Reflection and Insight Scale correlated with the respective single-item measure; IN, Insight subscale of the Self Reflection and Insight Scale correlated with the respective single-item measure; OT, Others' reflection subscale of the Self Reflection and Insight Scale correlated with the respective single-item measure*.

** *p < 0.01*.

* *p < 0.05*.

In summary, the results indicate that psychological literacy was best described in terms of three factors representative of generic graduate attributes, reflective processes, and the helping aspects of psychology as a profession, along with a stand-alone measure of interactional diversity. However, the indicators reflective of these factors were not consistent across data collected from different phases except in the case of the helping aspects of psychology as a profession. Single and multi-item measures of each facet were not found to be equivalent.

## Discussion

The main aim of our research was to begin the process of identifying and evaluating measures of psychological literacy dimensions. We identified existing multiple and single item measures for each of McGovern et al.'s (2010) nine facets of psychological literacy. Each of the identified multiple-item measures had acceptable internal reliability, and although confirmatory factor analyses identified deviations from good fit, the remaining psychometric properties of measures were deemed acceptable for the exploratory purposes of this research. The deviations from good model fit in each of the individual measure confirmatory factor analyses conducted suggested that further examination of the latent factor structures of each measure may be warranted in future research; however, this was beyond the scope of the current study, which focused on the latent factor structure *across* measures underlying psychological literacy.

A particular focus of our research was to examine the level of association between measures of the differing facets of psychological literacy, to determine whether the future development of measuring psychological literacy should focus on developing a unitary or multiple measures of psychological literacy. Correlations of subscales within measures were found to be moderate to high, while correlations between measures ranged from non-significant to moderate. The mixed magnitude of correlations between measures indicated the possibility of a higher order factor structure. Exploratory and confirmatory factor analyses on factor scores of measures suggested a higher order three factor solution with a stand-alone measure of interactional diversity. Consequently, the results from this research suggest that multiple measures of psychological literacy may be required to capture the complexity of psychological literacy as currently conceptualized.

The first of the three higher order factors represents Reflective Processes. The two reflection subscales (self and others) of the *Self-Reflection and Insight Scale* consistently loaded on this factor across factor analyses. The *Critical Thinking Disposition Scale* loaded with these subscales in the first sample only. Critical thinking and reflection are closely aligned concepts, with the combination sometimes referred to as “critical reflection” (e.g., Murray and Kujundzic, 2005), however the two constructs as measured in this research differ in terms of focus, with reflection focused on one's own and others' behavior and mental processes and criticial thinking focused on problem solving.

The second of the three higher order factors represents generic graduate attributes, which comprised significant loadings from the majority of the psychological literacy measures. Across samples, six measures consistently loaded on this factor: the *Need for Cognition Scale*, the *Information Literacy Self Efficacy Scale*, the *Self-Perceived Communication Competence Scale*, the *Integrity Scale*, the Insight subscale of the *Self-Reflection and Insight Scale* and the *Psychology Misconceptions Test*. An additional measure, the *Critical Thinking Disposition Scale*, loaded on this factor in the second sample only. These measures, with the exception of the *Psychology Misconceptions Test*, appear to tap into generic graduate attributes across disciplines, as reflected in University statements of graduate attributes (see, for example Barrie, 2007).

The third and final higher order factor represents psychology as a helping profession. The two sub-scales of the *Psychology as a Helping Profession* measure emerged as a distinct factor across all factor analyses. This scale, originally designed to measure undergraduate student beliefs about how studying psychology can aid personal growth and be applied to helping-related situations (Gervasio et al., 2010), was used as a measure of the fourth facet of psychological literacy: applying psychological principles to personal, social, and organizational issues in work, relationships, and the broader community. Gervasio et al. (2010) reported that students majoring in psychology scored significantly higher on this measure than students from non-psychology majors, adding support to the conceptualization of this dimension of psychological literacy as separate from generic graduate attributes. Further research is required to determine if this construct is what attracts students to study psychology, or something that is developed/learned as students progress through their degree in psychology.

The *Interactional Diversity Scale* did not significantly load on underlying factors in any of the factor analyses, and correlations with other psychological literacy measures were non-significant or low. This scale, providing a measure of student contact with other students from differing backgrounds, was used as a measure of the eighth facet of psychological literacy: recognizing, understanding, and fostering respect for diversity. The items in the measure focus on becoming acquainted and having serious discussions with students from a range of diverse backgrounds, and appear to provide a suitable self-report measure of this construct. The combination of low/non-significant correlations and failure to load on underlying psychological literacy factors suggests this psychological literacy dimension is distinct from other dimensions of psychological literacy.

The finding of a factor that appears to be tapping generic graduate attributes suggests the need for a reconsideration of what is meant by psychological literacy. Further research using subjective and objective measures of each of the psychological literacy dimensions administered across disciplines is required to determine whether some of the proposed facets of psychological literacy are generic graduate attributes, rather than psychology specific. If this were the case, we would expect similar scores on these measures across disciplines. This raises an interesting question for the future conceptualization of psychological literacy: should we discriminate between psychology-specific dimensions and those that are generic to undergraduate education across disciplines?

If the concept of psychological literacy is used to promote the value of the undergraduate psychology degree, an emphasis on facets that differentiate psychology graduates and graduates from other disciplines is warranted. Only a portion of psychology graduates continue on to work as psychologists. In Australia it has been estimated that 44% of fourth year students will follow vocational pathways other than professional psychology (Cranney et al., 2009). This figure is even higher in the UK with an estimated 80% of psychology graduates not progressing to professional psychologist status (Trapp et al., 2011). Articulating the specific unique skills and abilities psychology graduates bring to the workplace may help in the marketing of psychology graduates to a wide range of industries and employers.

Instead, if the term psychological literacy is used to convey the “total package” of skills and abilities of a psychology graduate, the inclusion of generic graduate attributes is warranted. Jones (2013) argues that graduate attributes are always embedded within specific cultural contexts, with the teaching and assessment of graduate attributes influenced by discipline. Perhaps what is needed here is the redefining of each of the generic graduate attributes in terms that are specific to the discipline of psychology.

The majority of measures used in this research are self-report measures. Only one objective test was included in the battery of measures: the *Psychology Misconceptions Test*. The other measures may be subject to the known limitations of student self-report data (Herzog and Bowman, 2011), including social desirability biases and poor recall. However, the limitations of self-report measures are frequently over-stated (Chan, 2009). While objective measures of behavioral constructs may be preferable where these are obtainable, some aspects of PL cannot be directly measured using behavioral measures (e.g., the second dimension of psychological literacy includes *valuing* the intellectual challenges required to use scientific thinking), and may be better captured by self-report measures (Chan, 2009).

Further, while some of the measures, such as the *Information Literacy Self-Efficacy Scale*, the *Self-perceived Communication Competence Scale*, and the *Interactional Diversity Scale* were clearly designed to be self-reports of ability or behavior, other measures were designed as trait rather than achievement measures. These include the *Critical Thinking Disposition Scale* and the *Need for Cognition Scale*. The *Critical Thinking Disposition Scale* is designed to measure disposition toward critical thinking (Sosu, 2013). However, the teaching of critical thinking skills should arguably result in higher ratings on many of the behavioral items in the measure (e.g., “I use more than one source to find out information for myself” and “I usually check the credibility of the source of information before making judgements”). The *Need for Cognition Scale* is designed to measure the “tendency to engage in and enjoy effortful cognitive endeavors” (Cacioppo et al., p. 306). While this measure taps an underlying preference for effortful cognitive engagement, previous research suggests that preference for this type of engagement may increase with years of tertiary education (Wang et al., 2014). Cross-sectional studies across years of study or longitudinal research are required to assess whether these dispositional measures of PL facets are sensitive to change over time in the psychology undergraduate population.

The second aim of our research was to compare single-item and multiple-item measures of each facet of psychological literacy. Based on the results found in this study, the single-item measures of each facet of psychological literacy were not strong analogs of their multiple-item counterparts. The strongest associations were found between measures of information literacy and communication competence, part of the generic graduate attributes. Weaker associations were found for measures underlying reflective processes and psychology as a helping profession.

This less-than-encouraging pattern of results suggests the need for further research in determining whether single-item or multiple-item measures best capture the facets of psychological literacy. The limited correlations between single- and multiple-item measures of each facet could be due to a mismatch in content covered by the multiple-item and single-item measures. The multiple-item measures were selected from available existing measures, and may not map exactly onto the facets of psychological literacy as described by McGovern et al. (2010). That is, the measures may have less than optimal construct validity. Future research is needed to develop and validate multi-item self-report measures specifically designed to measure each facet of psychological literacy. A further avenue for future research is to examine the predictive validity of single and multi-item measures.

As the title of the article suggests, we view this research as “a first approximation” in measuring psychological literacy. Further research is required on two fronts. First, based on the assumption that psychological literacy is something that is taught and learned during the undergraduate psychology degree, known groups' validity of these self-report measures of psychological literacy as something distinct to psychology should be tested. This can be achieved through a) comparing scores for psychology students and non-psychology students on each measure and b) comparing psychology students across years of study. If psychological literacy is something that is taught and learned in the undergraduate years, psychology students should score higher on each measure than students from other majors, and scores should increase with years of study of psychology. However, given the finding in the current research of what appears to be one generic graduate attributes factor and two factors that may be more specific to psychology, we would anticipate that differences between psychology and non-psychology students are likely to be smaller on measures subsumed in the generic graduate attributes factor than the other two factors, although we would predict that scores on all measures would increase across years of psychology education. Based on the results of known groups' validity testing, each of the measures can be re-assessed to determine their validity for future use in measuring psychological literature. The second recommended area of further research is the identification or development of objective measures of each of the nine facets of psychological literacy. This would enable objective testing of the development of psychological literacy across the years of the undergraduate psychology degree. It would also enable an assessment of the validity of self-report measures of psychological literacy as indicators of actual psychological literacy. In summary, the importance of psychological literacy within the context of undergraduate psychology training speaks to an imperative for further development of the measurement of the construct.

### Conflict of interest statement

The authors declare that the research was conducted in the absence of any commercial or financial relationships that could be construed as a potential conflict of interest.
